# Intrapulmonary Sequestration: A Rare Occurrence

**DOI:** 10.7759/cureus.8463

**Published:** 2020-06-05

**Authors:** Ghulam Aftab, Ankit Agrawal, Shashank Nuguru, Douglas Frenia

**Affiliations:** 1 Pulmonary Medicine, Saint Peter's University Hospital/Rutgers Robert Wood Johnson Medical School, New Brunswick, USA; 2 Internal Medicine, Saint Peter's University Hospital/Rutgers Robert Wood Johnson Medical School, New Brunswick, USA; 3 Pulmonary Critical Care, Saint Peter's University Hospital, New Brunswick, USA; 4 Pulmonary Critical Care, Saint Peter’s University Hospital, New Brunswick, USA

**Keywords:** pulmonary sequestration

## Abstract

Pulmonary sequestration is a rare occurrence. Here, we present a case of a 45-year-old female who, on CT scan of the chest, was found to have a left lower lobe consolidation. Despite antibiotic treatment, the consolidation remained persistent. On repeat imaging with CT scan with contrast, it was found that the consolidation was pulmonary sequestration. The patient was referred to cardiothoracic surgery to remove pulmonary sequestration through video-associated thoracoscopic surgery.

## Introduction

Pulmonary sequestration was first described by Pryce in 1946 in the Journal of Pathology and Bacteriology [1]. Pulmonary sequestration is a rare congenital malformation of the respiratory tract. It constitutes approximately 0.15%-6.4% of all congenital pulmonary malformations [2]. It is defined as a non-functioning mass of parenchymal lung tissue that lacks communication with the tracheobronchial tree and is supplied by an anomalous systemic artery. Anatomically, it is classified as intralobar sequestration, where it is located within a normal lobe without its own visceral pleura, or extralobar sequestration, which is outside the normal lung with its own visceral pleura. Here, we present a case of a 45-year-old female who had a diagnosis of intrapulmonary sequestration [3].

## Case presentation

**Figure 1:**
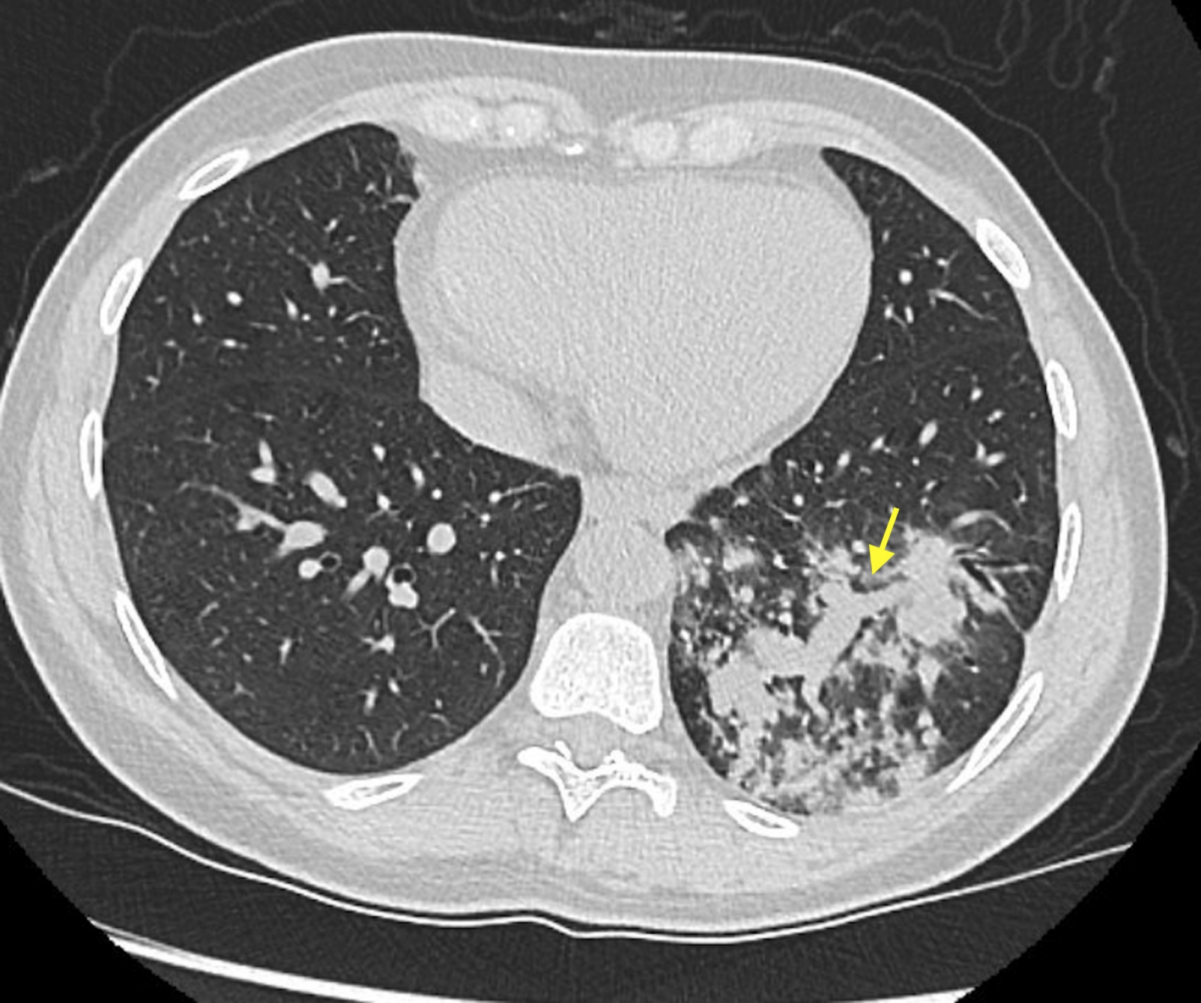
Transverse view of the CT chest showing left lower lobe consolidation (yellow arrow).

A 45-year-old Caucasian female presented with a left-sided breast mass. An excisional biopsy showed a high-grade phyllodes tumor, which was treated by resection. Preoperatively, a chest radiograph was obtained, which revealed a left lower lobe shadow suspicious of consolidation. At the time, this was considered to be a community-acquired pneumonia though she had no symptoms. She was hemodynamically stable. Laboratory investigations were within normal limits. She was subsequently treated with a seven-day course of antibiotics. Follow-up chest radiograph showed persistence of the infiltrate which led to a non-contrast computed tomography (CT) scan of the chest. It revealed a moderate consolidation of the left lower lobe and hilar lymphadenopathy (Figure 1).

**Figure 2:**
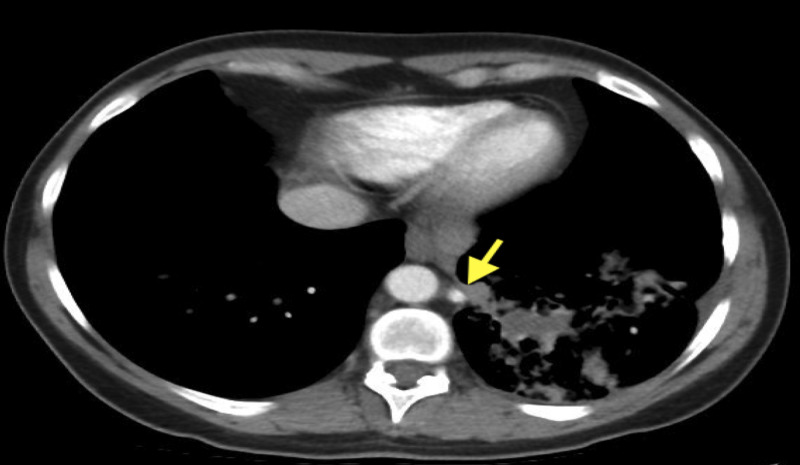
CT chest with contrast. The yellow arrow shows feeding artery entering area of consolidation.

**Figure 3:**
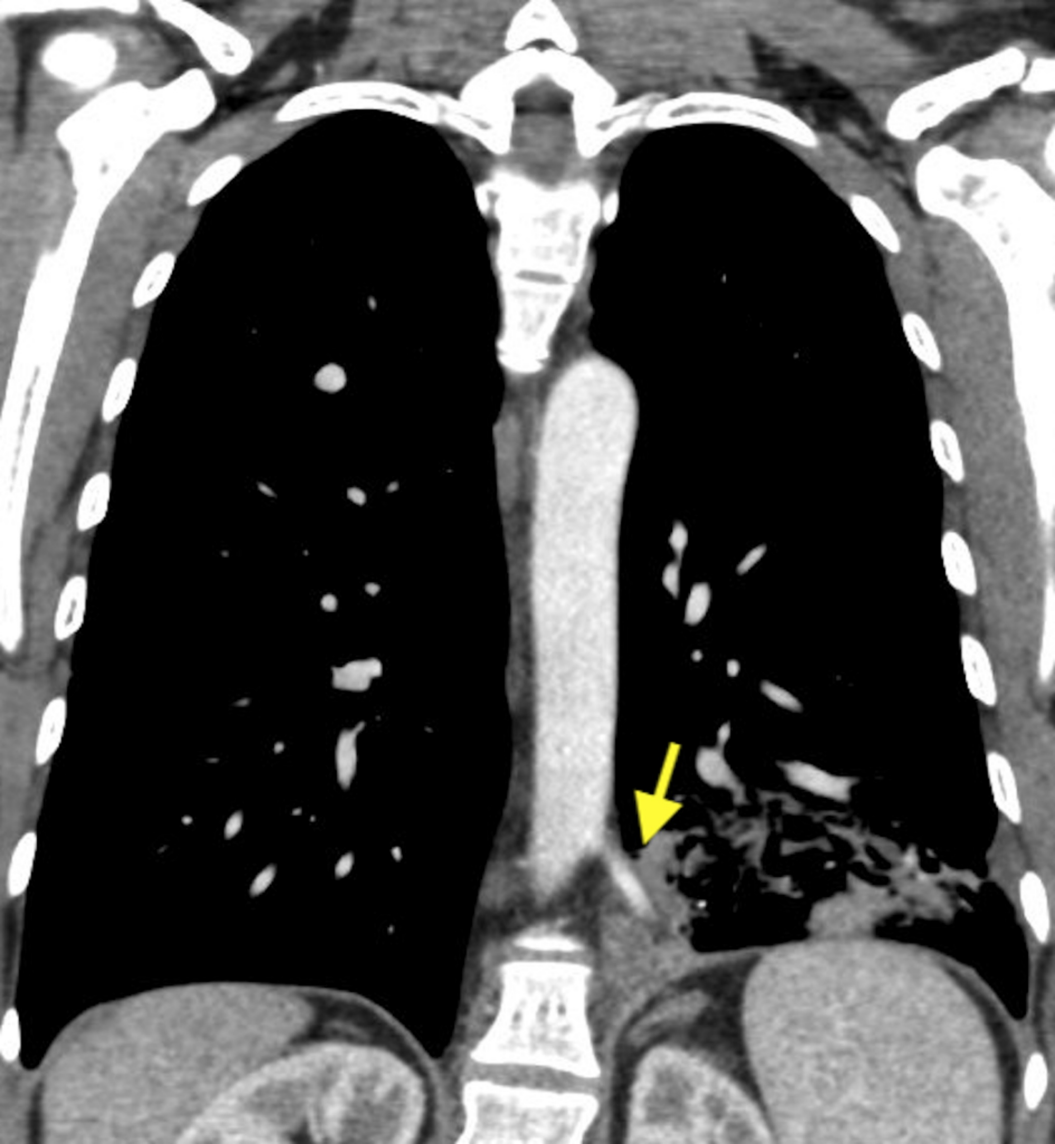
Coronal view of the CT chest with contrast. The yellow arrow shows the feeding artery entering the area of infiltration.

She, again, denied any signs of infection including sputum production, hemoptysis, shortness of breath or cough. She also denied a history of frequent pulmonary infections. She was a former smoker and had a 20-pack year history. A fiberoptic bronchoscopy was conducted for her unresolving left lower lobe infiltrate. Brushing of the area of infiltration was obtained along with the lymph node biopsy, which turned out to be non-malignant. At this juncture, a chest CT scan with contrast was obtained, which showed an artery from descending aorta feeding the area of infiltration highly suggestive of pulmonary sequestration (Figures 2, 3).

After discussion with the patient, it was decided to pursue a resection. She was referred to a cardiothoracic surgeon for a video-assisted thoracoscopic surgery (VATS) lobectomy procedure. The patient underwent left lower lobectomy. Pathology report after lobectomy confirmed the features consistent with an intralobar sequestration.

## Discussion

Pulmonary sequestration is a rare congenital malformation of dysplastic lung tissue. The sequestered lung does not communicate with the tracheobronchial tree and is supplied by an anomalous systemic arterial source, most commonly from the aorta [4]. Intralobar sequestration is four times more common than the extralobar type [5]. Diagnosis during adulthood is relatively uncommon as 60% of cases are diagnosed in the first decade of life and is a rarity in adults greater than 40 years of age [6]. 

Most patients develop symptoms in infancy or early childhood. Symptoms may be non-specific, including cough, chest pain and shortness of breath. Some patients develop recurrent pneumonias and bronchiectasis [7]. Approximately 15% of patients remain asymptomatic [8]. Our patient was asymptomatic and had denied previous pulmonary infections.

As in our patient, sequestration occurs mostly in the left hemithorax, in the posterior basal segment of the left lower lobe [8,9]. In 75% of the patients, the supply to the intralobar sequestration is from the descending thoracic aorta [9].

CT angiography scan is the imaging test of choice as it can show the anomalous artery feeding into the pulmonary sequestration [4,9]. Non-contrasted CT imaging is sometimes adequate to aid in the diagnosis of sequestration. However, in our case, the diagnosis was not clear until CT angiography was performed. Pulmonary angiography is considered to be the gold standard; however, it is not commonly used as the diagnosis is mostly clinched on CT scan imaging.

On cut section, the intralobar pulmonary sequestration represents mucus filled airways and small cysts which may be filled with purulent material. On histological examination, there is mucus stasis in the airways and a systemic artery accompanies the airways [10].

Surgical resection is considered the treatment of choice for intralobar sequestration especially in symptomatic patients [8,11,12]. Even in asymptomatic patients, surgical resection is often recommended due to the risk of serious future complications, including infections, massive hemoptysis and malignant transformation [2,13]. The usual surgery is lobectomy either via VATS or standard thoracotomy. Our patient had a VATS lobectomy performed.

## Conclusions

Pulmonary sequestration is an uncommon finding especially in the adult population. Imaging modalities, especially contrast-enhanced CT scan, can aid in diagnosis by localizing the aberrant arterial blood supply to the sequestered lung parenchyma. Contrasted CT scan is also important for preoperative evaluation in these patients.
